# Altered profile of circulating microparticles in nonvalvular atrial fibrillation

**DOI:** 10.1002/clc.23158

**Published:** 2019-02-20

**Authors:** Panjaree Siwaponanan, Rassamon Keawvichit, Suthipol Udompunturak, Saowalak Hunnangkul, Kanit Reesukumal, Kasama Sukapirom, Kovit Pattanapanyasat, Rungroj Krittayaphong

**Affiliations:** ^1^ Division of Cardiology, Department of Medicine, Faculty of Medicine Siriraj Hospital Mahidol University Bangkok Thailand; ^2^ Siriraj Center of Research Excellence in Microparticle and Exosome in Diseases, Department of Research and Development, Faculty of Medicine Siriraj Hospital Mahidol University Bangkok Thailand; ^3^ Department of Clinical Pathology, Faculty of Medicine Siriraj Hospital Mahidol University Bangkok Thailand; ^4^ Division of Clinical Epidemiology, Department of Research and Development Faculty of Medicine Siriraj Hospital, Mahidol University Bangkok Thailand

**Keywords:** cellular origin, circulating microparticles (cMPs), Nonvalvular atrial fibrillation

## Abstract

**Background:**

Nonvalvular atrial fibrillation (AF) is the most common cardiac arrhythmia, and it is associated with the prothrombotic state. Circulating microparticles (cMPs) are membrane vesicles that are shed from many cell types in response to cell activation and cell apoptosis. Several studies reported that cMPs may play a role in the hypercoagulable state that can be observed in patients with AF. The aim of this study was to determine the levels of total cMPs and characterize their cellular origins in AF patients.

**Methods:**

Atotal of 66 AF patients and 33 healthy controls were enrolled. This study investigated total cMP levels and their cellular origin in AF patients using polychromatic flow cytometry.

**Results:**

AF patients had significantly higher levels of total cMPs (median 36.38, interquartile range [IQR] 21.16‐68.50 × 10^5^ counts/mL vs median 15.21, IQR 9.91‐30.86 × 10^5^ counts/mL; *P* = 0.004), platelet‐derived MPs (PMPs) (median 10.61, IQR 6.55‐18.04 × 10^5^ counts/mL vs median 7.83, IQR 4.44‐10.26 × 10/mL; *P* = 0.009), and endothelial‐derived MPs (EMPs CD31+ CD41−) (median 2.94, IQR 1.78‐0.60 × 10^5^ counts/mL vs median 1.16, IQR 0.71‐2.30 × 10^5^ counts/mL; *P* = 0.001) than healthy controls after adjusting for potential confounders. Phosphatidylserine positive MP (PS + MP) levels were similar compared between AF patients and healthy controls.

**Conclusion:**

The results of this study revealed a marked increase in total cMP levels, and evidence of elevated endothelial damage and platelet activation, as demonstrated by increased PMP and EMP levels, in AF patients. Additional study is needed to further elucidate the role of cMPs (PMPs and EMPs) in the pathophysiology of and the complications associated with AF.

## 1 INTRODUCTION

Nonvalvular atrial fibrillation (AF), the most common sustained cardiac arrhythmia, is associated with an approximately 5‐fold increase in the risk of developing ischemic stroke.^1^ The prevalence of AF increases with age, and is more common in men than women.^2^, ^3^ The risk factors for stroke in AF patients include congestive heart failure, hypertension, age ≥ 75, female gender, diabetes mellitus, vascular disease, and previous stroke.^4^

Microparticles (MPs) belong to the family of extracellular vesicles, and they are shed from the plasma membranes of activated or apoptotic cells. MPs are defined by their size, ranging from 100 to 1000 nm in diameter.^5^ MPs were thought to play a crucial role in hemostasis and thrombosis.^6^ The procoagulant properties of MPs are corroborated by their involvement in the increased risk of thromboembolic events in various diseases, including thalassemia,^7^ atherosclerosis,^8^ and venous thrombosis.^9^ Endothelial‐derived MP (EMP) levels have been reported to impact the severity, lesion volume, and outcome of acute ischemic stroke.^10^ Platelet‐derived MP (PMP) levels have been proposed as a potent marker for cerebral injury in ischemic stroke.^11^ Elevated levels of procoagulant MPs, like PMPs and EMPs, have been observed in persistent and permanent AF, which suggests that sustained and enhanced production of circulating procoagulant MPs may reflect a prothrombotic state with an increased risk of atrial thrombosis and thromboembolism.^12^, ^13^, ^14^ However, no study has investigated the role of MPs in the pathophysiology of AF. Accordingly, the aim of this study was to use polychromatic flow cytometry to investigate total circulating MP (cMP) levels and their cellular origin in AF. The results of this study may yield important clues regarding the association between cMPs and the thrombogenicity and pathophysiology of AF.

## 2 METHODS

### 2.1 Study population

This study was conducted at the Division of Cardiology, Department of Medicine, Faculty of Medicine Siriraj Hospital, Bangkok, Thailand. The protocol for this study was approved by the Siriraj Institutional Review Board (SIRB) (COA no. Si 199/2017), and complied with the principles set forth in the Declaration of Helsinki (1964). All study participants provided written informed consent prior to inclusion.

Sixty‐six AF patients (28 with paroxysmal AF, 7 with persistent AF, and 31 with permanent AF) and 33 healthy individuals were recruited. All subjects underwent thorough historical investigation and 12‐lead electrocardiography to confirm cardiac rhythm. The exclusion criteria were refusal of provide consent, history of myeloproliferative disorders, thrombocytopenia, ischemic stroke within the previous 3 months, malignancy, rheumatic mitral valve disease, acute infectious or inflammatory disease, and pregnancy. Control subjects had no cardiovascular risk factors, no history of atrial arrhythmias, no clinical evidence of disease, and no current cardiovascular treatment. Controls also underwent electrocardiography to document sinus rhythm.

### 2.2 Creatinine assessment

Serum was used to analyze creatinine levels using an automatic biochemistry analyzer (Roche Cobas 8000 modular analyzer Series, Roche Diagnostics GmbH, Mannheim, Germany).

### 2.3 Circulating microparticle determination

Serum was obtained from clotted whole blood, and then centrifuged at 1500*g* for 15 minutes to remove all cells. We modified the method from Choudhury et al study which determined microparticle levels by flow cytometry from one centrifugation step plasma.^12^ Aliquots of each centrifuged serum sample were immediately subjected to polychromatic flow cytometric analysis. Twenty‐five microliters of each serum sample was incubated with appropriate monoclonal antibodies (mAbs) in 300 μL of filtered Annexin‐V Binding Buffer (BD Biosciences, Franklin Lakes, New Jersey) for 15 minutes at room temperature in the dark. The staining panels of mAbs for enumeration of cMPs were modified from previous study.^15^ Flow cytometric gating strategy for circulating microparticles analysis are shown in Figure S1. Data analysis was performed using FlowJo software version 10 (FlowJo, LLC, Ashland, Oregon). The absolute count, expressed as number of cMPs per mL, was obtained by dividing the number of positive cMP events or cell‐derived MP events by the number of bead events, and then multiplying that figure by TC bead concentration. Red blood cells derived microparticles (RMP) were detected by CD235a or glycophorin‐A.

### 2.4 Statistical analysis

All data analyses were performed using PASW Statistics version 18 (SPSS, Inc., Chicago, Illinois). Kolmogorov‐Smirnov test was used to evaluate the distribution of data. Since circulating MP levels were not normally distributed, comparisons between groups were performed using Mann‐Whitney *U* test. Kruskal‐Wallis test was used to compare independent samples from more than two groups. cMP levels were expressed as log‐transformed counts per mL (log MPs/mL). Unpaired *t* test was used to compare normally distributed continuous variables. Proportion comparisons were performed using *χ* ^2^ test. Analysis of covariance (ancova) was used to adjust for potential confounders. MP levels were transformed on a logarithmic scale before entering ancova analysis. Data are presented at number and percentage or mean ± SD. All statistical tests were two‐tailed, and a *P*‐value less than 0.05 was considered statistically significant.

## 3 RESULTS

### 3.1 Patient Characteristics

The baseline characteristics of 66 AF patients and 33 control subjects are summarized in Table 1. Significant differences were observed for age (*P* < 0.0001), gender (*P* = 0.001), and body mass index (BMI) (*P* < 0.0001) between AF patients and controls. Worsening of renal function was presented by increasing creatinine levels in AF patients. Baseline characteristics of AF patients are shown in Table 2.

**Table 1:** Baseline characteristics of AF patients and healthy controls

Variable	AF patients (n = 66)	Healthy controls (n = 33)	*P*‐value
Age (years)	70.2 ± 10.7	56.5 ± 3.8	***<0.001***
Male gender, n (%)	45 (68.2%)	10 (30.3%)	***0.001***
BMI (kg/m^2^)	26.3 ± 4.7	23.1 ± 2.6	***<0.001***
SBP (mm Hg)	126.9 ± 18.0	123.7 ± 14.3	0.376
DBP (mm Hg)	73.6 ± 13.5	77.0 ± 10.5	0.210
Heart rate, beats per 1 minute	76.95 ± 15.0	73.21 ± 16.2	0.258
Creatinine (mg/dL)	1.17 (0.98‐1.40)	0.81 (0.70‐0.92)	***<0.001***

Abbreviations: AF, atrial fibrillation; BMI, body mass index; SBP, systolic blood pressure; DBP, diastolic blood pressure.

Data presented as mean ± SD and 25% to75% interquartile range unless otherwise indicated.

A *P*‐value<0.05 indicates statistical significance.

**Table 2:** Medical history and medication use in AF patients

Medical history	n (%)
Ischemic stroke	15 (22.7%)
Hypertension	56 (84.8%)
Coronary artery disease	13 (19.7%)
Congestive heart failure	14 (21.1%)
Diabetes mellitus	20 (30.3%)
Dyslipidemia	47 (71.2%)
Bleeding history	14 (21.2%)
Systemic embolism	2 (3.0%)
PE or DVT	3 (4.5%)
Hyperthyroidism	6 (9.1%)
Hypothyroidism	2 (3.0%)
Medications	
Beta‐blocker	48 (72.7%)
Digoxin	5 (9.3%)
Statin	46 (69.7)
Dihydropyridine CCB	26 (39.4%)
ACEI or ARB	33 (50.0%)
Proton pump inhibitor	11 (16.67%)
Aspirin	21 (31.8%)
Warfarin	52 (78.8%)

Abbreviations: ACEI, angiotensin‐converting enzyme inhibitor; AF, atrial fibrillation; ARB, angiotensin II receptor blockers; CCB, calcium channel blocker; DVT, deep vein thrombosis; PE, pulmonary embolism.

### 3.2 cMP levels and their cellular origins analysis

**Figure 1:**
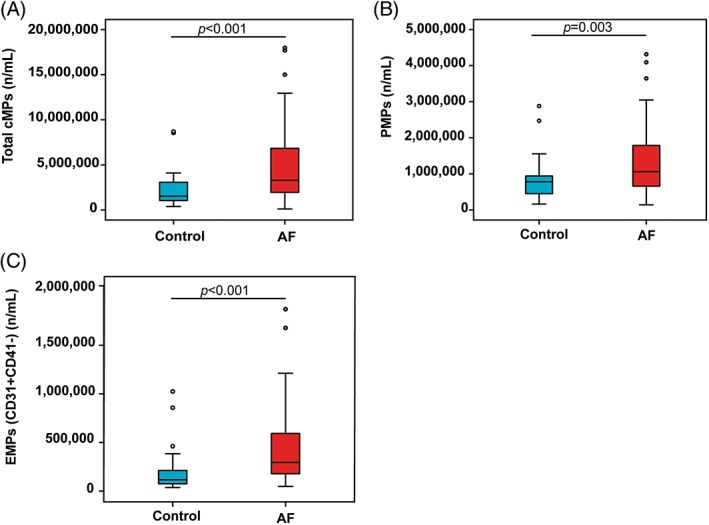
Levels of (A) total cMP, (B) PMP, and (C) EMP (CD31+ CD41−) in the nonvalvular AF and control groups are presented as box plots. The middle line indicates the median. The top and bottom of each box indicates the 25th and 75th percentiles. (AF, atrial fibrillation; cMPs, circulating microparticles; EMPs, endothelial‐derived microparticles; PMPs, platelet‐derived microparticles)

The cellular origins of cMPs in peripheral blood were identified by staining cMPs with cell‐specific surface markers. PMPs were the most abundant population of cMPs in both AF patients and healthy controls. AF patients had significantly higher levels of total cMPs (*P* < 0.001), PMPs (*P* = 0.002), EMPs (CD31+ CD41−) (*P* < 0.001), red blood cell‐derived microparticle (RMPs) (*P* = 0.020), and tissue factor bearing microparticle (TF‐MPs) (*P* = 0.014) than healthy controls. There were no significant differences in the levels of EMPs (CD144+), leukocyte‐derived microparticle (LMPs), or phosphatidylserine expressing microparticle (PS + MPs) between AF patients and healthy controls (Table 3). ancova was performed to examine for differences in cMPs between groups after adjusting for potential confounders. After adjusting for age, gender, and BMI, the total cMP, PMP, and EMP (CD31+ CD41−) levels were still higher in the AF group than in the control group. Comparisons of total cMP, PMP, and EMP (CD31+ CD41−) levels between AF patients and control subjects are shown in Figure 1. No significant differences were observed for the level of any type of MP among paroxysmal, persistent, and permanent AF patients.

**Table 3:** Comparison of cMP levels between AF patients and controls

MPs	Healthy controls (n = 33)	AF patients (n = 66)	*P*‐value	Adjusted *P*‐value^a^
Total cMPs	6.22 ± 0.32	6.54 ± 0.41	***<0.001***	***0.004***
PMPs	5.83 ± 0.82	6.03 ± 0.30	***0.002***	***0.009***
PS+MPs	5.23 ± 0.42	5.13 ± 0.35	0.234	0.880
LMPs	4.47 ± 0.24	4.53 ± 0.29	0.336	0.839
EMPs (CD144+)	5.17 ± 0.35	5.18 ± 0.32	0.898	0.607
EMPs (CD31+ CD41a−)	5.12 ± 0.35	5.50 ± 0.34	***<0.001***	***0.001***
RMPs	5.25 ± 0.36	5.42 ± 0.32	***0.020***	0.138
TF‐MPs	4.67 ± 0.32	4.86 ± 0.36	***0.014***	0.145

Abbreviations: AF, atrial fibrillation; cMPs, circulating microparticles; EMPs, endothelial‐derived microparticles; PMPs, platelet‐derived microparticles; PS + MPs, phosphatidylserine expressing microparticles; LMPs, leukocyte‐derived microparticles; RMPs red blood cell‐derived microparticles; TF‐MP, tissue factor bearing microparticles).

Data presented as mean ± SD unless otherwise indicated.

A *P*‐value<0.05 indicates statistical significance.

MP levels are expressed as log‐transformed counts per mL (log MPs/mL).

a Analysis of covariance (ancova) was used to adjust for age, gender, and BMI.

### 3.3 Effect of cardiovascular risk factors and anti‐thrombotic therapy on cMP levels

Advanced age, male gender, hypertension, diabetes mellitus, dyslipidemia, and previous stroke are cardiovascular risk factors that have been reported to be associated with elevated cMP levels in AF patients. To assess the influence of these factors on increased cMP, PMP, and EMP (CD31+ CD41−) levels in AF patients, we evaluated each of these parameters in AF patients compared between the presence or absence of each cardiovascular risk factor and antithrombotic drugs. Our results revealed no significant differences in total cMP, PMP, or EMP (CD31+ CD41−) levels relative to any of the evaluated risk factors or antithrombotic drugs (Figures S2 and S3 ).

## 4 DISCUSSION

Our polychromatic flow cytometric analysis showed circulating MPs levels in nonvalvular AF patients. To the best of our knowledge, this is the first study in AF patients to demonstrate increased levels of total cMPs. PS+ is used as a marker for apoptotic cells, and is frequently used in the analysis of cMPs; however, not all cMPs express PS. As such, the study of only PS + MPs may lead to a misunderstanding of the role of cMPs in disease. cMPs also express markers from their parental cells, such as CD41 for platelets, CD235a for erythrocytes, and CD45 for leukocytes. The study of cMP cellular origin may provide insight into the pathophysiology of AF.

In this study, total cMP levels were significantly higher in AF patients than in healthy controls even after adjusting for potential confounders. Moreover, total cMP levels did not differ among AF patients relative to cardiovascular risk factors or the type of antithrombotic treatment. Of note, most cMPs (90%) in healthy individuals were characterized using our antibody panel, whereas about 45% of cMPs in the AF group were not able to be characterized. This implies that there are other unknown cellular origins of cMPs in the blood of AF patients. We postulate that increased total cMP levels may result from the pathophysiology of AF, as the atrium is composed of many cell lineages, including endothelial cells, fibroblasts, and cardiomyocytes. Evidence of cellular apoptosis was found in animal and human hearts with AF, including abnormal myocytes, a high degree of DNA breakage, strong caspase‐3 (CASP‐3) expression, and decreased anti‐apoptotic BCL‐2 proteins.^16^ The possible roles of apoptosis and cell activation in MP generation have been described in AF, possibly through low shear stress, hypoxia, stretch, inflammation, and oxidative stress in the atrium, the combination of which promotes cell apoptosis and the release of MPs.^2^

We found higher EMP levels (CD31+ CD41−) in AF patients than in healthy controls. It is known that EMPs play a pivotal role in vascular homeostasis. Several stimuli, such as shear stress, inflammation, proapoptotic stimulation, and vascular damage, have been described as promoting EMP secretion.^17^ EMP levels are suggested to be a potential marker of endothelial dysfunction.^18^ Increased EMP levels have been reported in the presence of cardiovascular risk factors, including hypertension,^19^ diabetes,^20^ and dyslipidemia.^21^ In this study, we also observed EMP levels in AF patients who had a history of several underlying risk factors, and we found no differences in these levels relative to any specific cardiovascular risk factors. This is probably the result of our patients being appropriately medicated to control their underlying diseases, as evidenced by the normal blood pressures observed in our AF patients. Several studies have demonstrated that MPs play role in vascular function through endothelial modulator regulation; nitric oxide (NO) and endothelin‐1 (ET‐1).^22^, ^23^ AF induces a loss of organized atrial contraction with turbulent blood flow and decreased shear stress in the atrium. This mechanism resulted in downregulation of NO synthase, which leads to decreased endothelial cell survival.^24^ Thus, elevated EMP levels in AF may result from endothelial damage.^18^, ^19^, ^20^, ^21^ On the other hand, elevated EMP levels may contribute to endothelial dysfunction in AF.^22^, ^23^ in vitro study demonstrated that ET‐1 stimulates the release of EMP from human umbilical vein endothelial cells (HUVECs).^25^ Elevated ET‐1 levels have been reported in AF patients.^26^ Another possible mechanism of increased EMP levels in AF may result from ET‐1 stimulation.

The levels of CD31+ EMPs and CD144+ EMPs appear to increase in apoptotic endothelial cells and have reported as the potential biomarkers for risk assessment of endothelial dysfunction. In our study, we found the increased levels of CD31+ CD41− EMP in AF patients, whereas there was no difference in the levels of CD144+ EMP between AF patients and controls. This contrast may be because of different sites of endothelial injury, noting that PECAM‐1 (CD31) is located mainly outside of the endothelial cell adherens junction, whereas VE‐cadherin (CD144) is localized at the adherens junction.^27^ CD62E+ EMP levels are increased in activated endothelial cells. Clinical studies have shown that elevated CD62E+ EMP levels indicate a hypoglycemic event after insulin induction in diabetes status.^28^ Increased CD62E+ EMP levels are observed in AF patients after treatment by digoxin, suggesting the use of digitalis in AF is associated endothelial activation.^29^ Moreover, EMP levels have been shown the potential relationship with worsening renal dysfunction in AF patients with chronic kidney disease (CKD), as presented by a significant negative correlation with estimated glomerular filtration rate (eGFR) and a positive correlation with serum creatinine.^30^

High levels of PMPs were observed in AF patients with no observed differences among the different types of AF, which is consistent with previous finding.^12^ That study found PMP levels to be significantly higher in both AF patients and disease controls when compared with healthy controls. However, they found no significant difference between AF patients and disease control subjects. The authors of that study suggested that high PMP levels are related to the underlying cardiovascular diseases, but not to AF.^12^ Although we did not investigate PMP levels in disease control subjects with risk factors, we did compare the levels of PMP between AF patients with and without cardiovascular risk factors. The results revealed no significant differences in PMP levels between these two AF groups. However, the small sample size of our AF subsets limits the power of our findings. It is possible that the antiplatelet and antihypertensive drugs used by our study patients may have modulated the PMP levels in their respective cardiovascular diseases, as reported in other studies.^31^, ^32^ Acetylsalicylic acid (ASA) have shown to reduce PMP levels in patients with coronary artery disease.^33^ in vitro study, ASA have been reported that inhibit the PMP generation in platelet‐rich plasma from healthy subjects are induced by platelet agonists.^34^

PMPs are generated from activated platelets. Platelet activation is observed in AF as represented by increase β‐thromboglobulin levels.^35^, ^36^ However, there is limited evidence indicating that it directly enhances thrombotic risk in AF.

**Figure 2:**
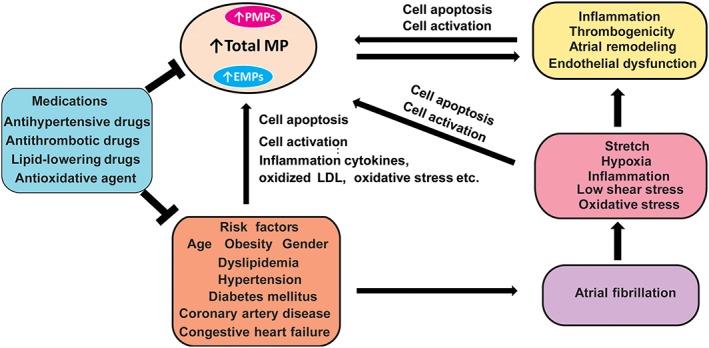
Putative mechanism of the relationship between microparticle (MP) generation and atrial fibrillation ( EMPs, endothelial‐derived microparticles; LDL, low‐density lipoprotein cholesterol; PMPs, platelet‐derived microparticles)

In normal conditions, cells release MPs to maintain homeostasis, and this contributes to the regulation of several physiological processes. Relative to the pathophysiology of disease, changes in the number, composition, and function of MPs are thought to promote hypercoagulability, proinflammation, and endothelial dysfunction as part of the development of cardiovascular diseases.^37^ AF and the effect of cardiovascular risk factors may lead to an increase in total MP levels, especially PMPs and EMPs. During AF, low shear stress, oxidative stress, hypoxia, stretch, and inflammation promote cell apoptosis and cell activation, which leads to MP generation.^2^ Increased levels of PMPs and EMPs have been observed in uncontrolled hypertension, diabetes mellitus, and dyslipidemia, as well as in some CVDs, such as coronary artery diseases and congestive heart failure. Moreover, MPs may promote the pathophysiology of AF. MPs may play a role in the development of thrombosis, inflammation, and reduced NO production, which in turn leads to endothelial dysfunction. MPs that are shed from apoptotic cells harboring functional metalloproteinase contribute to proteolysis of the fibrillary matrix, which results in plasmin generation and tissue remodeling. Some medications, such as antihypertensive, antithrombotic, lipid‐lowering, and antioxidant drugs, which are used to control risk factors can also modulate PMP and EMP levels (Figure 2).

Phosphatidylserine‐expressing MPs play an important role in thrombosis, because they provide a catalytic surface to support the assembly of blood coagulation factors. Ederhy et al reported increased procoagulant MP (PS+MP) levels in AF, which suggests the presence of AF as a strong predictor of procoagulant MPs. They suggested that PS+MP may reflect a hypercoagulable state in AF.^13^ This is consistent with a recent study by Wang et al found that PS+MP levels to be significantly higher in nonvalvular AF patients than in controls with risk factors. The levels of PS+MP in AF with left atrial thrombi were also significantly higher than in those without thrombi.^14^ However, both the immediately aforementioned studies excluded patients who were treated with anticoagulant and/or antiplatelet therapy to avoid the effect of these drugs on cMP release and their procoagulant activity. Unlike above mentioned studies, our study found no difference in the PS+MP levels between AF patients and controls. It seems likely that our AF patients did not manifest hypercoagulable state, and this may have been because of drug modification of PS+MP levels. It was reported that antiplatelet and antihypertensive treatment can reduce the amounts of circulating procoagulant MP.^38^, ^39^

AF patients were receiving warfarin (78.8%) and aspirin (31.8%) to prevent the ischemic stroke. The effect of warfarin and aspirin on MP levels have demonstrated in AF in which there was no difference in PMP levels between receiving therapy with aspirin and warfarin.^12^ This observation is consistent with our data; we found that no effect of warfarin and aspirin on cMP levels and all cellular origins. Nowadays, non‐vitamin K antagonist oral anticoagulants (NOAC) have been approved as alternatives for warfarin in patients with nonvalvular AF. The effect of the traditional oral anticoagulant, warfarin, and NOAC on TF‐MP levels in AF was investigated. This study showed that there was no significant difference in TF‐MP levels between AF patients treated with warfarin and those treated with NOAC. The authors suggested that the use of traditional or newer anticoagulants, prothrombotic biomarkers are still generated at increased levels in patients with AF.^40^

Although the reduction of protein C and protein S reflects the high consumption of activated protein C (APC) and was associated with an increase in MP levels in some disease state,^41^ the relation of reduction in APC and protein S during warfarin treatment and MP levels is still unclear. Moreover, APC has been demonstrated to induce the release of microparticle‐associated EPCR.^42^

Moreover, we found RMP and TF‐MP levels to be significantly higher in AF patients than in controls, but these differences did not persist after adjusting for potential confounders. These results suggest that RMP and TF‐MP levels are more related to age, gender, and BMI than to the AF itself. The number of RMPs was reported to correlate positively with age in women, but not in men.^43^ Correlations were identified between the numbers of various TF‐positive MP subpopulations and body mass index in type 2 diabetes.^44^ LMPs play an important role in both proinflammatory and anti‐inflammatory processes. In our study, we found LMP levels not to be significantly different between AF patients and controls, which suggesting that LMPs may not be the main effector of inflammation in AF. It remains to be determined whether the above findings are also observed if the control subjects have cardiovascular risk factors.

## 5 CONCLUSION

This is the first report that found a marked increase in total cMP levels in AF patients. The evidence of elevated endothelial damage and platelet activation were demonstrated by increased PMP and EMP levels in AF patients. Additional study is needed to further elucidate the role of cMPs (PMPs and EMPs) in the pathophysiology and the complications associated with AF.

## CONFLICTS OF INTEREST

The authors declare no potential conflict of interests.

## Supporting information


**FIGURE S1** Flow cytometric gating strategy for the analysis of circulating microparticles. R1 is the gate for all events. (A) The cMP gate was established based on size, using standard calibration beads sized 220, 580, 750, and 1300 nm. (B) cMP are circulating microparticle gate. TC represents TruCOUNT beads gate. The cellular origins of microparticles were identified using cell type‐specific surface markers. (C) PS + MPs were determined by FITC‐Annexin V positive population. (D) PMPs were identified using BV421‐CD41 positive population. (cMPs, circulating microparticles; PMPs, platelet‐derived microparticles; PS + MPs, phosphatidylserine positive microparticles)

**FIGURE S2** Effect of cardiovascular risk factors on total cMP, PMP, and EMP (CD31+ CD41−) levels among AF patients

**FIGURE S3** Effect of age gender and antithrombotic drugs on total cMP, PMP, and EMP (CD31+ CD41−) levels among AF patients
