# Supplementary figures and images for: Altered profile of circulating microparticles in nonvalvular atrial fibrillation
Source: Clin Cardiol. 2019 Feb 20;42(4):425–31. doi: 10.1002/clc.23158 (PMC6712324; doi:10.1002/clc.23158)

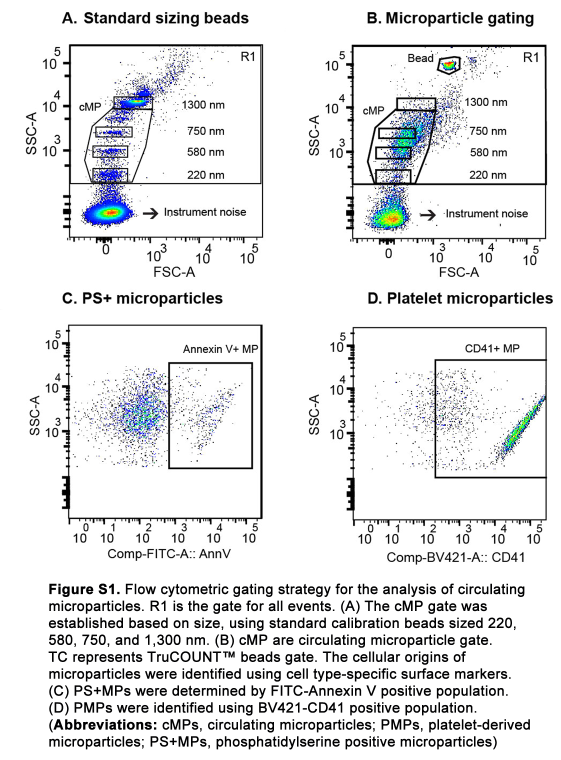

Supplement: Supplementary file 1 — FIGURE S1 Flow cytometric gating strategy for the analysis of circulating microparticles. R1 is the gate for all events. (A) The cMP gate was established based on size, using standard calibration beads sized 220, 580, 750, and 1300 nm. (B) cMP are circulating microparticle gate. TC represents TruCOUNT beads gate. The cellular origins of microparticles were identified using cell type‐specific surface markers. (C) PS + MPs were determined by FITC‐Annexin V positive population. (D) PMPs were identified using BV421‐CD41 positive population. (cMPs, circulating microparticles; PMPs, platelet‐derived microparticles; PS + MPs, phosphatidylserine positive microparticles) [file CLC-42-425-s001.tif]

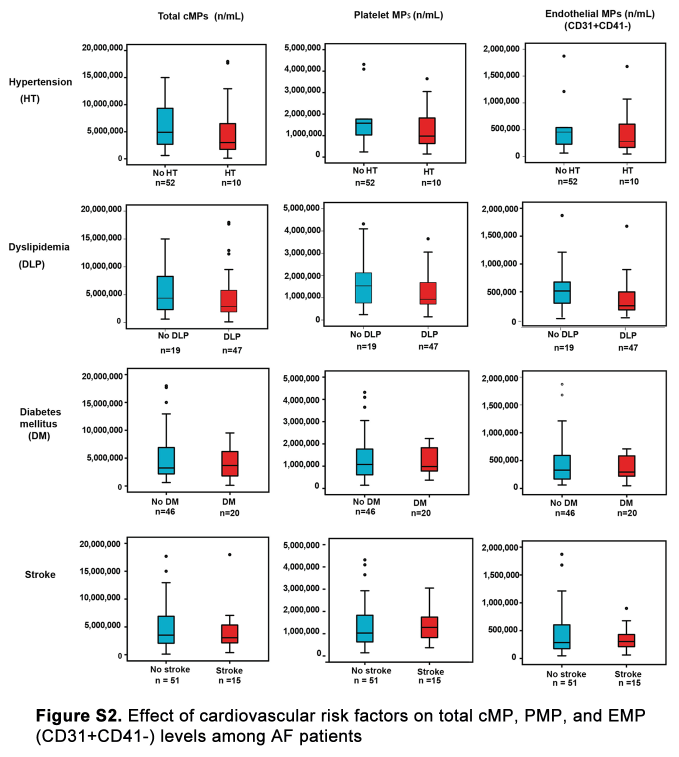

Supplement: Supplementary file 2 — FIGURE S2 Effect of cardiovascular risk factors on total cMP, PMP, and EMP (CD31+ CD41−) levels among AF patients [file CLC-42-425-s002.tif]

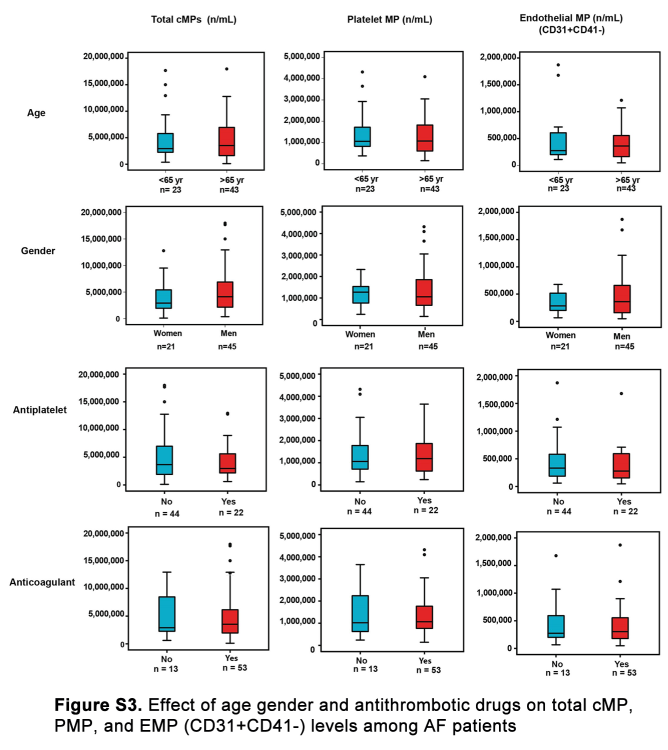

Supplement: Supplementary file 3 — FIGURE S3 Effect of age gender and antithrombotic drugs on total cMP, PMP, and EMP (CD31+ CD41−) levels among AF patients [file CLC-42-425-s003.tif]
